# Environmental regionalization and endemic plant distribution in the Maghreb

**DOI:** 10.1007/s10661-021-09707-6

**Published:** 2022-01-15

**Authors:** Łukasz Walas, Asma Taib

**Affiliations:** 1 Institute of Dendrology Polish Academy of Sciences, Parkowa 5, 62-035 Kórnik, Poland; 2 Ecole Nationale Supérieure Agronomique, El Harrach, Algeria

**Keywords:** Biogeography, Environmental clusters, Endemic plants, Algeria, Morocco, Tunisia

## Abstract

Clustering methods based on environmental variables are useful in the planning of conservation strategies for species and ecosystems. However, there is a lack of work on the regionalization of the vast space of North Africa and the distribution of plant species. The current lists of endemic plants are focused mostly on an occurrence at the country level and not on regions with different conditions. The aim of this work was to lay out an environmental scheme for northwest Africa and to collect data about the occurrence of endemic plants in this area. Clustering with 12 of 33 tested environmental rasters was performed to divide the Maghreb into environmental clusters. Then, a list of 1618 endemic plant taxa (1243 species and 375 subspecies) was prepared and their distribution in estimated environmental clusters was examined. Eleven clusters with different conditions were estimated. The main drivers of regionalization were temperature amplitude, precipitation seasonality, and precipitation of the warmest quarter. According to the occurrence of endemic plants, northwest Africa may be divided into three zones: Atlas, Mediterranean (two environmental clusters), and southern zone (eight environmental clusters). The presented results provide a good basis for understanding the spatial patterns of the Maghreb, including its environment and species diversity. A designed list of endemic plant species together with environmental data may facilitate the planning of future research in north Africa and arranging methods of biodiversity protection.

**Supplementary information:**

The online version contains supplementary material available at 10.1007/s10661-021-09707-6.

## Introduction

Environmental regionalization allows one area to be divided into smaller regions with similar conditions (Long et al., 2010). Understanding spatial patterns in the environment, together with the description of the flora and fauna in particular regions, is crucial for conservation planning of endangered taxa and whole ecosystems (Coops et al., 2009; Leathwick et al., 2003; Pressey et al., 2000). Regionalization is especially useful in the case of highly heterogeneous areas, which are characterized by diverse types of environments because borders between particular regions could be very clear. A good example is northwest Africa, where many types of ecosystems, including mountainous forests, shrublands, deserts, oases, and wetlands, occur. Various types of vegetation are connected with different bioclimatic zones: humid and semi-arid areas in the north, and large, extremely dry areas in the south (Boukri, 2017; Nouaceur et al., 2013). The entire Maghreb region, consisting of Algeria, Morocco, and Tunisia, can be divided into three main environmental areas: the northern Mediterranean part, southern dry part, and mountainous region of Atlas.

The northern part, characterized by mild, wet winters and hot, dry summers, hosts a great abundance of endemic plant species (Véla & Benhouhou, 2007). It is part of the Mediterranean “hotspot,” which is one of the most diverse areas in terms of vegetation; moreover, it is the third most important hotspot for floristic diversity and endemism (Valderrábano et al., 2018). This region encompasses around 25,000 species of vascular plants, of which almost half are considered endemic (Fois et al., 2017; Valderrábano et al., 2018). The coastal area of Maghreb reflects the great biodiversity of the whole Mediterranean Basin; 878 endemic plant species were described in Morocco (Rankou et al., 2013), 290 in Algeria (Benhouhou et al., 2018; Dobignard & Chatelain, 2010–2013), and 26 species and 13 subspecies in Tunisia (Valderrábano et al., 2018).

The high peaks of the Atlas mountains are much cooler than the rest of North Africa. These mountains separate coastal, humid areas from the southern, dry part; they also retain much of the moisture that comes in from the Atlantic Ocean. The natural vegetation in this area consists of forests that transition to scrub and grassland formations at higher elevations. The mountainous areas of Atlas and Rif, characterized by a suitable climate with high precipitation, are especially rich in terms of plant species (Bouchet et al., 2018). Areas of Middle and High Atlas are important centers of biodiversity (Médail & Diadema, 2009).

The southern part of Maghreb is different from the suitable north—the largest warm desert in the world, the Sahara, is located in this dry region with very poor vegetation (Brito et al., 2014). The vast area of the Sahara experiences periodic transitions from dry to wet conditions, which is associated with the occurrence of the monsoons. Such a climate shift occurs approximately every 20,000 years (Born et al., 2008; Skonieczny et al., 2019; Williams et al., 2016). Climatic transitions have an impact on the flora, which during dry periods is shifted to more suitable areas like the Mediterranean coast, Atlas, and the mountainous regions inside the Sahara, such as Ahaggar (Boucheneb & Benhouhou, 2012). The present, dry environment formed around 2700 cal yr B.P (Kröpelin et al., 2008). Despite the harsh conditions in the current Sahara, around 2800 species of vascular plants occur there, from which even 25% may be endemic (Le Houérou, 2009). In the future, conditions in this region probably will change again, which may cause further shifts in species occurrence (Pausata et al., 2020).

The flora of North Africa is still rather poorly described, especially in comparison with other regions around the Mediterranean Sea (Fenu et al., 2014; Georghiou & Delipetrou, 2010; Lobo et al., 2001; Médail et al., 2019). However, several works were carried out to create a detailed list of the plant species. The first synthesis on the flora of the Maghreb was “Flore de l’Afrique du Nord” by René Maire (Maire, 1952). A complete taxonomic reference for all parts of North Africa has been recently published in the “Index synonymique de la flore d’Afrique du Nord” (Dobignard & Chatelain, 2010–2013), which may be treated as a source for up-to-date nomenclature because it is very complete and precise (Valderrábano et al., 2018). An important source for the flora of Algeria is the “Nouvelle flore de l’Algérie et des régions désertiques méridionales” by Quézel and Santa (1962) and for Tunisian flora “Catalogue synonymique commenté de la Flore de Tunisie” by Le Floch et al. (2010). However, these works are focused on the country level, not on environmental regions. Thus, it is difficult to understand the pattern of occurrence of particular species, especially endemic taxa with a limited range.

Endemics have a very high priority in the conservation system (Fois et al., 2017) because they are responsible for a big part of biodiversity (Estill & Cruzan, 2001). Endemism is difficult to define because whether a species is endemic depends strongly on which and how large an area is considered - one species may be endemic to only a small region, while another may be endemic to an entire continent (Thompson, 2005; Hobohm, 2014). Although a small range is not necessary for a species to be considered endemic, many important taxa are limited range endemics, occurring in small areas with suitable conditions. Such taxa are usually threatened because of a low tolerance to changes in the environment. Several factors shape the distribution of species, like topography, temperature, precipitation, and human influence (Abdelaal et al., 2020; Brito et al., 2014; Fenu et al., 2014; Morrone, 2018). Predicted, fast climate change may exceed the capacity of endemic species to adapt or shift their range; the situation is worsened by the growing human impact (Abdelaal et al., 2020; Fois et al., 2017; Taib et al., 2020). Thus, the extinction risk of many plants endemic for North Africa will increase in the near future due to unfavorable changes in environmental conditions, connected with higher temperatures and decreasing precipitation (Valderrábano et al., 2018; Taib et al., 2020). Paleondemites are particularly endangered, being relict species often associated with small climatic refugia (Thompson, 2005). Implementing necessary conservation actions for species and ecosystems will require an understanding of the historical and current distribution of the flora, as well as its ecology, genetic variation, and demography (Valderrábano et al., 2018).

Biogeography, as a science that attempts to describe and explain spatial patterns of biological diversity, helps to better understand species distribution and is useful in conservation planning (Abdelaal et al., 2020; Fenu et al., 2014). The Maghreb is a vast space that is ecologically vulnerable (Taabni & Jihad, 2012) but little studied in the case of environmental heterogeneity and plant species distribution. Most of the literature is focused on separate countries of the region, but there is a lack of broader studies covering both the Mediterranean and arid zones. Thus, the aim of the present work was to lay out a comprehensive environmental scheme for countries of northwest Africa based on a detailed and versatile dataset of environmental layers. Additionally, this study aimed to collect data about the occurrence of plants endemic to the Maghreb, identify the most endemic-rich regions, and explore the relationships among the estimated environmental units.

## Materials and methods

### Study area

The Maghreb (from Arabic *al-Maghrib*, “the west”) is a region in Northwest Africa. In the narrower sense, this area includes the territory of Algeria, Morocco, and Tunisia; in a broader sense, it also includes Libya, Western Sahara, and Mauritania. In our work, we focused on the Maghreb in a narrower sense. This region covers 2,991,901 km^2^, including 2,381,741 km^2^ of Algeria, 446,550 km^2^ of Morocco, and 163,610 km^2^ of Tunisia. The highest point is Toubkal in the Atlas Mountains (4,167 m a.s.l), and the lowest point is Sebkha Tah in Morocco (− 55 m a.s.l.). The entire Maghreb can be divided into three environmental areas. The first consists of the main chain of the Atlas Mountains; this region is cold and humid (annual precipitation circa 390 mm, annual mean temperature 13.7 °C). The second region consists of northern, coastal areas, which have a Mediterranean climate according to the Köppen-Geiger climate classification (Beck et al., 2018). This region is humid and warm (annual precipitation circa 518 mm, annual mean temperature 17.5 °C). The biggest part of Maghreb, however, is dry and hot (annual precipitation circa 78 mm, annual mean temperature 23.1 °C), and dominated by the vast area of the Sahara desert.

### Environmental clustering

Several environmental variables (33) were tested during the study (Table S1)—19 bioclimatic variables from the Chelsa database (https://chelsa-climate.org, Karger et al., 2017); altitude raster from Global Multi-resolution Terrain Elevation Data 2010 datasets (https://topotools.cr.usgs.gov/gmted_viewer/viewer.htm, Danielson & Gesch, 2011); two soil rasters (pH and carbon content at a depth 5–15 cm) from the SoilGrids website (https://soilgrids.org, Hengl et al., 2014); four rasters of habitat heterogeneity (Shannon diversity of Enhanced Vegetation Index (EVI), Range of EVI, Evenness of EVI, Coefficient of variation of EVI) from EarthEnv (https://www.earthenv.or, Tuanmu & Jetz, 2015); insolation raster (yearly average global irradiance on a horizontal surface) from the PVG project website (https://ec.europa.eu/jrc/en/pvg, Huld et al., 2012); and additional climatic data (six rasters: aridity index, continentality, moisture index, annual potential evapotranspiration, potential evapotranspiration seasonality and Emberger coefficient) from the ENVIREM dataset (https://envirem.githu, Title & Bemmels, 2018). All rasters were resampled into the same resolution (2515 × 1859 pixels, about 1 km^2^ each).

To detect the correlation between variables, variance inflation factors (VIFs) were calculated using the *vif* function from package usdm in R (Naimi, 2015). In each iteration of the procedure, one variable with the highest value of VIF was excluded, and VIFs were calculated again for the remaining rasters. This procedure was repeated until all remaining variables had a VIF below 5. Finally, 12 factors were selected for the next steps of analysis (Table 1).

**Table 1:** List of the variables used in clustering

Variable	Source	Code	Unit	VIF
Mean temperature of wettest quarter	Chelsa	Bio8	°C	2.33
Precipitation seasonality	Chelsa	Bio15	%	2.33
Altitude	GMTED	Alt	m	2.56
Precipitation of warmest quarter	Chelsa	Bio18	mm	2.62
Coefficient of variation of EVI	EarthEnv	VEG_Coef		2.69
Potential evapotranspiration seasonality	ENVIREM	PET	mm/month	3.32
Mean diurnal range of temperature	Chelsa	Bio2	°C	3.44
Soil pH in H_2_O	SoilGrids	Soil_pH		3.47
Organic carbon content in soil	SoilGrids	Soil_Carbon	mg/m^2^	3.77
Mean temperature of driest quarter	Chelsa	Bio9	°C	3.88
Sun irradiation	PVG	Sun	W/m^2^	4.28
Shannon diversity of EVI	EarthEnv	VEG_Shan		4.96

To choose the appropriate number of geographic clusters, the function *fviz_nbclust* with the silhouette method from the package factoextra in R was used (Kassambara & Mundt, 2017). Clustering based on selected variables was carried out in SAGA GIS software, according to the *K-mean Clustering for Grids* and *Hill-Climbing method* (SAGA, 2013). A *Majority filter* was used to remove small artifacts (single pixels of one cluster inside another) from the output raster. Estimated environmental clusters were compared with the Ecoregions 2017^©Resolve^ map, which is a revisited version of the terrestrial ecoregions map (Olson et al., 2001), and with the Köppen-Geiger climate classification map (Beck et al., 2018).

The significance of variables within clusters was estimated by the lowest shared value (Abdelaal et al., 2020). This parameter is the ratio of variable range within the cluster and range within the whole region of Maghreb. Variables that were the most important during the clustering process showed the lowest shared values. For each cluster, the mean values of the 33 tested variables were calculated (Table S1). According to normalized values of variables, agglomerative hierarchical clustering was performed using the function *hclust* in R to estimate environmental similarities between particular environmental clusters.

### Endemic plant taxa

A list of endemic plant taxa (species and subspecies, Table S2) occurring in the Maghreb area (Algeria, Morocco and Tunisia) was created according to the data available in databases (*African Plant Database*, 2021; *eflora Maghreb,* 2021; *Flore du Maroc*, 2021; *GBIF*, 2021; *North Africa Trees*, 2021; *Plants of the World online,* 2021; *The Gymnosperm Database*, 2021; *The IUCN Red List of Threatened Species,* 2021) and the literature (Quézel & Santa, 1962; Baum and Rajhathy (1976); Pottier-Alapetite, 1979–1981; Navarro & El Oualidi, 1997; Benabid & Cuzin, 1997; Neffati et al., 1999; Ben El Mostafa et al., 2001; Médail et al., 2001; Upson & Jury, 2002; Besnard et al., 2007; Dobignard & Chatelain, 2010–2013; Le Floch et al., 2010; El Oualidi et al., 2012; Salemkour et al., 2012; Yahi et al., 2012; Alonso et al., 2013; Hamel et al., 2013; Rankou et al., 2013, 2015; Miara et al., 2014, 2017, 2018; Sękiewicz et al., 2015; El Mokni et al., 2015; Bouzabata et al., 2016; Véla et al., 2016; Bouchibane et al., 2017; Ghrabi-Gammar et al., 2017; Barberá et al., 2018; Bouchet et al., 2018; Bouahmed et al., 2019; El Mokni & Peruzzi, 2019; Lauterbach et al., 2019; Moukrim et al., 2019; Djelid et al., 2020; Gabriel et al., 2020; Taib et al., 2020). The taxonomic nomenclature was revised according to “Index synonymique de la flore d’Afrique du Nord” (Dobignard & Chatelain, 2010–2013). The list of taxa was transformed into a binary matrix of absence-occurrence with taxa names in columns and environmental clusters in rows. This matrix was used to calculate distance matrix with dist function in R. Subsequently, hierarchical clustering was performed using the function *hclust* in R. The resulting dendrogram was compared with the results of hierarchical clustering for environmental variables. Function *indval* with 1000 randomizations from labsdv package was used to perform indicator value analysis (Dufrêne & Legenre, 1997). This analysis estimated for which class (cluster) each species had the highest indicator value and which had the probability of obtaining a value over the specified iterations that would be as high as the indicator value. The endemic species densities were compared using *α*-index sensu Hobohm and the Arrhenius equation (Arrhenius, 1921; Hobohm, 1998), which is commonly used for this purpose (Fois et al., 2020; Hobohm, 2003).

## Results

### Environmental clusters

**Fig. 1:**
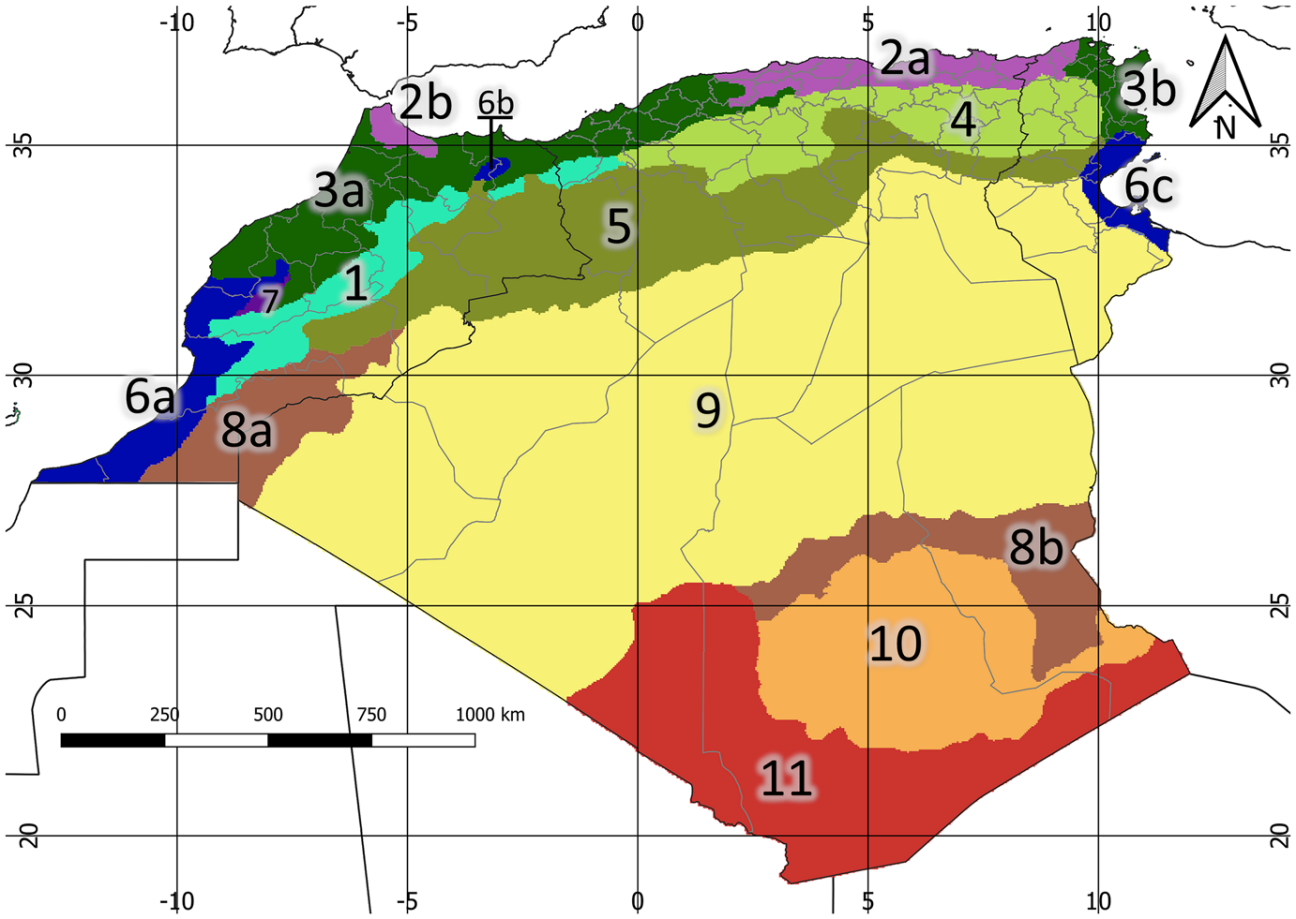
Clusters and subclusters of Maghreb according to K-mean clustering with K = 11. Environmental clusters: 1–Atlas, 2–Numidian-Rifian (2a–Numidian, 2b–Rifian), 3–Mediterranean (3a–Atlanto-Mediterranean, 3b–Carthaginian), 4–Cirtaic, 5–Saharan Atlas, 6–Tachelchit-Gabesian (6a–Tachelhit, 6b–Taurirt, 6c–Gabesian), 7–Marrakeshan, 8–Tassilio-Saharan (8a–Western Saharan, 8b–Tassilian), 9–Central Saharan, 10–Hoggarian, 11–Tanezzouft

The highest value of the average silhouette width was obtained for *K* = 3, with a visible peak for *K*= 11 (Fig. S1). However, analyses for a few clusters would not be informative for such a large area because *K* = 3 showed only simple division into the oceanic, dry, and mountainous zones. Thus, for further analyses, *K* = 11 was chosen. Such a number of geographical clusters allowed the division of a large area of Maghreb into clusters that had different conditions but avoided creating several small areas or few large zones. Some of the environmental clusters consisted of distant subclusters that shared similar environmental conditions. The following clusters were distinguished (Fig. 1): 1–Atlas, 2–Numidian-Rifian (2a–Numidian, 2b–Rifian), 3–Mediterranean (3a–Atlanto-Mediterranean, 3b–Carthaginian), 4–Cirtaic, 5–Saharan Atlas, 6–Tachelchit-Gabesian (6a–Tachelchit, 6b–Taurirt, 6c–Gabesian), 7–Marrakeshan, 8–Tassilio-Saharan (8a–Western Saharan, 8b–Tassilian), 9–Central Saharan, 10–Hoggarian, 11–Tanezzouft.

The most important factors according to the shared percentage of variables were mean diurnal temperature range (most significant factor for the separation of regions 1, 10, and 11, Table 2), precipitation seasonality (main factor for clusters 2, 3, 4, and 5), and precipitation in the warmest quarter (the most important factor for regions 6, 7, 8, and 9; Fig. S2).

**Table 2:** The shared values of variables used in the analysis. The most important variables (< 30%) are bolded. Codes of variables as in Table 1

**Variable**	**Region**											
		1	2	3	4	5	6	7	8	9	10	11
PET		53%	50%	74%	36%	36%	76%	**28%**	64%	43%	**28%**	37%
VEG Coef		70%	99%	78%	44%	100%	56%	47%	65%	78%	47%	49%
VEG Shan		99%	76%	100%	100%	100%	99%	89%	98%	99%	82%	89%
Sun		75%	60%	69%	43%	53%	63%	**22%**	32%	43%	**23%**	**23%**
Soil Carbon		63%	78%	87%	56%	36%	74%	**24%**	**28%**	40%	**18%**	**21%**
Soil pH		55%	75%	83%	67%	34%	42%	**30%**	44%	**25%**	**23%**	**17%**
Altitude		86%	57%	48%	53%	70%	48%	**16%**	46%	32%	57%	**29%**
**bio2**		**26%**	50%	82%	**26%**	**25%**	68%	**6%**	**22%**	43%	**17%**	**14%**
bio8		56%	37%	51%	63%	75%	47%	**15%**	70%	66%	61%	63%
bio9		75%	43%	49%	77%	57%	44%	**14%**	87%	47%	93%	79%
**bio15**		31%	**18%**	**23%**	**18%**	**21%**	34%	**8%**	34%	70%	36%	80%
**bio18**		32%	42%	38%	87%	51%	**22%**	**4%**	**16%**	**19%**	47%	**28%**

### Endemic plant taxa and hierarchical clustering

The created list consists of 1618 endemic taxa. The Maghreb was divided according to the occurrence of endemic plants into three main zones (Fig. 3A): Atlas Mountains (Atlas cluster), northern coastal areas with a mild climate (Numidian-Riffian and Mediterranean clusters), and interior with southern parts of seacoasts (remaining eight clusters). These zones had a similar number of endemic taxa, although they were different sizes. The Atlas zone, consisting of only one cluster (1), has 79,644.70 km^2^ and 781 endemic taxa. The Atlas cluster is the coldest area in the Maghreb and hosts unique biodiversity, especially in the Middle and High Atlas mountains. It has the highest value of the *α*-index (2.14), which indicates the high density of endemic taxa (Table S3).

The Mediterranean part of the Maghreb has an area of 269,859.85 km^2^ and 940 endemic plants. Two clusters forming this zone (2 and 3) are rich in the case of endemic plant species; in the Numidian-Rifian–occur 572 taxa (330 in Numidian subcluster, 298 in Rifian subcluster), and in the Mediterranean cluster 678 (in Atlanto-Mediterranean subcluster 617, in Carthaginian subcluster 91). The Numidian-Rifian cluster is the wettest area in North-West Africa; it is also characterized by rich soils. The Mediterranean cluster has similar conditions; however, it has a more continental climate and poorer soils. Values of *α*-index in these northern regions are high (with exception of Carthaginian subcluster, Table S3).

The southern zone of Maghreb, consisting of eight clusters (4–11), has 2,652,043.01 km^2^ and 702 endemic taxa. The Cirtaic cluster is a mountainous area, which shows environmental similarities with Atlas, but is drier and hosts much fewer plant species. South of it is the Saharan Atlas cluster, covered by dry woodlands and steppes, which separate the northern, humid areas from the Sahara. The Tachelchit-Gabesian cluster consists of areas close to the sea but characterized by low precipitation; these areas are located in southern Morocco, southern Tunisia, and the valley of the Moulouy River. On the border of three main zones (Atlas, Mediterranean, and south) is the small Marrakeshan cluster with only 40 endemic taxa. This low biodiversity is connected with a very small area of this cluster (Fig. S3). Another border area is the Tassilio-Saharan cluster, which borders Sahara on the south-east and west. This cluster has intermediate conditions between the arid desert and more humid mountainous areas. The hyper-arid area of Sahara is covered by the Central Saharan cluster, which is the biggest environmental cluster in the Maghreb; vegetation in this area is very limited, however, the value of *α*-index (0.86) is higher than in Tassili and Gabesian subclusters (0.38 and 0.55, respectively), as well as in the Hoggarian cluster (0.67). Although the mountainous area of the Hoggarian cluster is well known as the refugium, we found only 30 endemic taxa there. The small number of species may have been related to the lack of research on this isolated area. Additionally, many species occurred both in these mountains and in Tibesti in north Chad; such subendemic species were excluded from our analyses. In the deep south of Algeria is the Tanezzouft cluster. No taxa endemic for the Maghreb region were found in this region; thus, this area was excluded from the analyses. A low number of endemic species in the south part of Maghreb could be the result of a lack of data and poor description of this remote area in the literature.

**Fig. 2:**
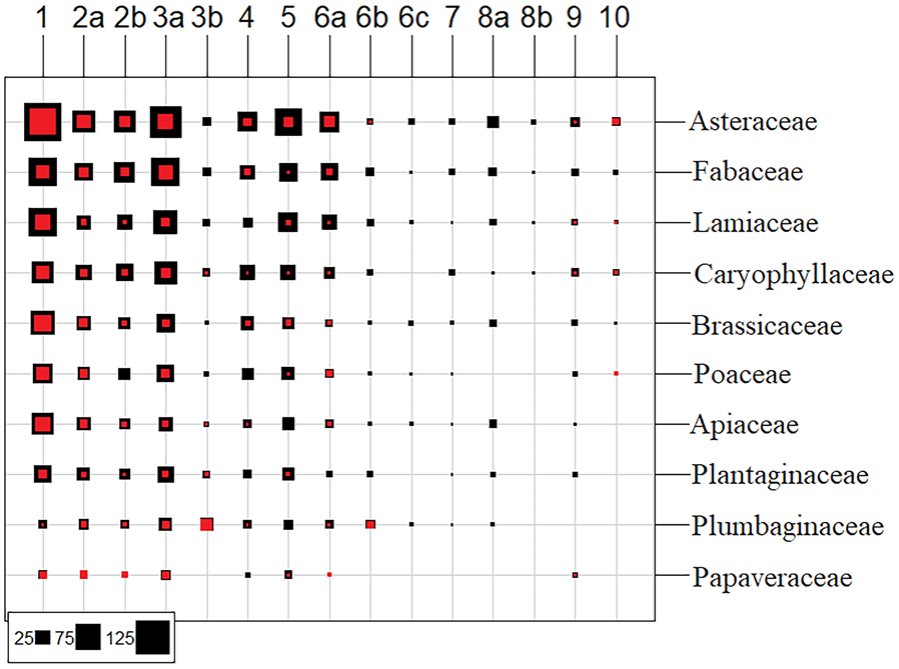
The number of taxa in ten families with the higher number of endemics in North Africa, according to clusters and subclusters. Black squares show numbers of all taxa, red squares show numbers of taxa that occur only in a particular cluster or subcluster. Environmental clusters: 1–Atlas, 2–Numidian-Rifian (2a–Numidian, 2b–Rifian), 3–Mediterranean (3a–Atlanto-Mediterranean, 3b–Carthaginian), 4–Cirtaic, 5–Saharan Atlas, 6–Tachelchit-Gabesian (6a–Tachelhit, 6b–Taurirt, 6c–Gabesian), 7–Marrakeshan, 8–Tassilio-Saharan (8a–Western Saharan, 8b–Tassilian), 9–Central Saharan, 10–Hoggarian

Asteraceae was the largest family in the case of endemic taxa in the Maghreb (309 taxa, Fig. 2), followed by Fabaceae (170), Lamiaceae (152), Caryophyllaceae (113), and Brassicaceae (103). Thirty-one genera occurred only in the analyzed region of Maghreb (Table S4)—12 from the Brassicaceae family; 8 from family Asteraceae; 2 per family in Amaranthaceae, Apiaceae, and Fabaceae; and 5 genera from other families.

Many plant species were distributed both in North Africa and the Iberian Peninsula; during our investigation, more than 350 taxa with such spatial patterns of distribution were found, for example, *Quercus canariensis* Willd. An interesting example of a subendemic species is *Tetraclinis articulata* (Vahl) Mast., which is typical for North Africa, but also has relic stands in Malta and the Iberian Peninsula, near Cartagena (Sánchez-Gómez et al., 2013).

**Fig. 3:**
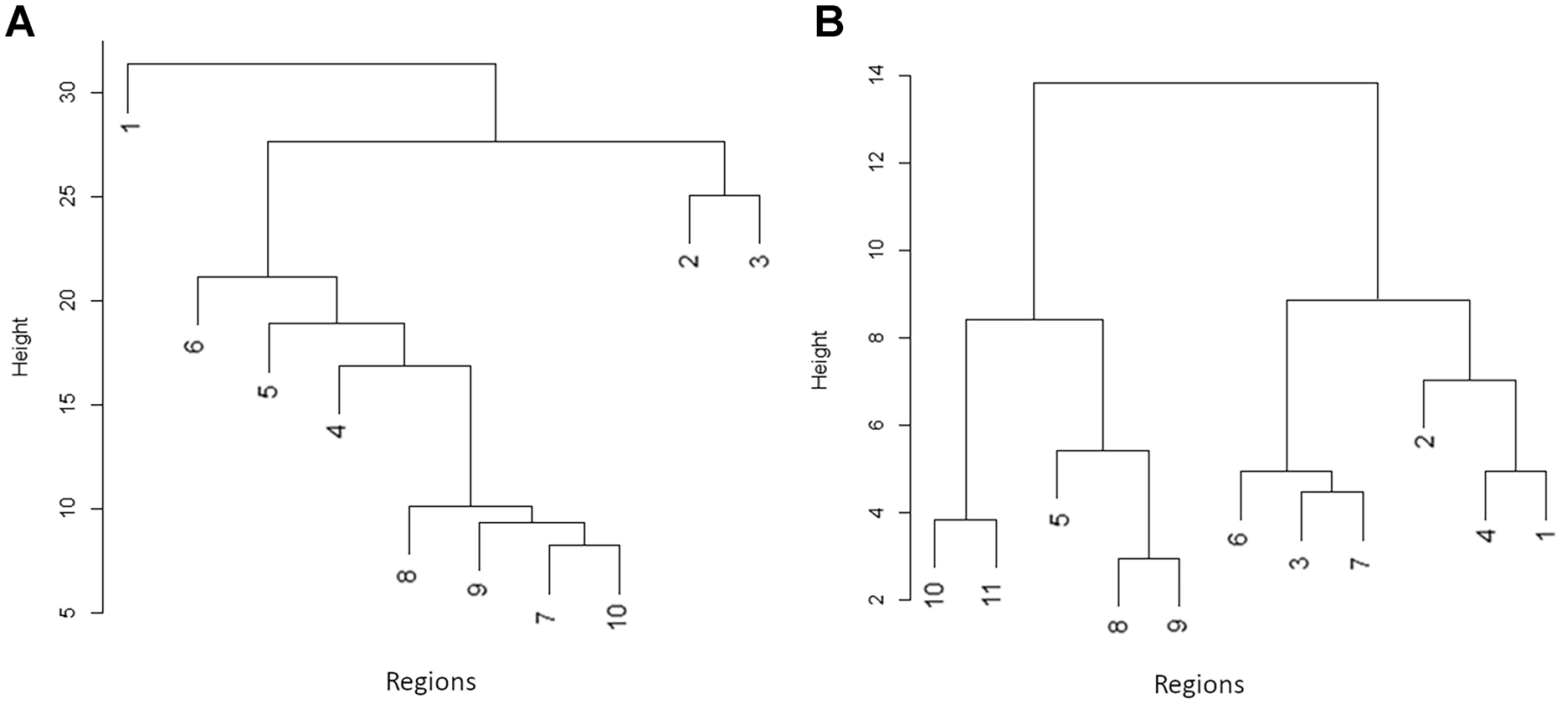
Results of hierarchical clustering according to occurrence of endemic plant taxa (A) and environmental variables (B). Environmental clusters: 1–Atlas, 2–Numidian-Rifian, 3–Mediterranean, 4–Cirtaic, 5–Saharan Atlas, 6–Tachelchit-Gabesian, 7–Marrakeshan, 8–Tassilio-Saharan, 9–Central Saharan, 10—Hoggarian, 11–Tanezzouft

Clustering according to endemic plants showed similarity between the Numidian-Rifian and Mediterranean clusters (Fig. 3A). However, in the case of environmental variables, the Mediterranean cluster was more similar to Tachelchit-Gabesian and Marrakeshan clusters. Atlas was distant from all other clusters in terms of endemic taxa but had similar environmental conditions to the Cirtaic and Numidian-Rifian clusters (Fig. 3B). All dry and hot southern clusters (8–11), together with the Saharan Atlas, formed a third environmental group.

**Fig. 4:**
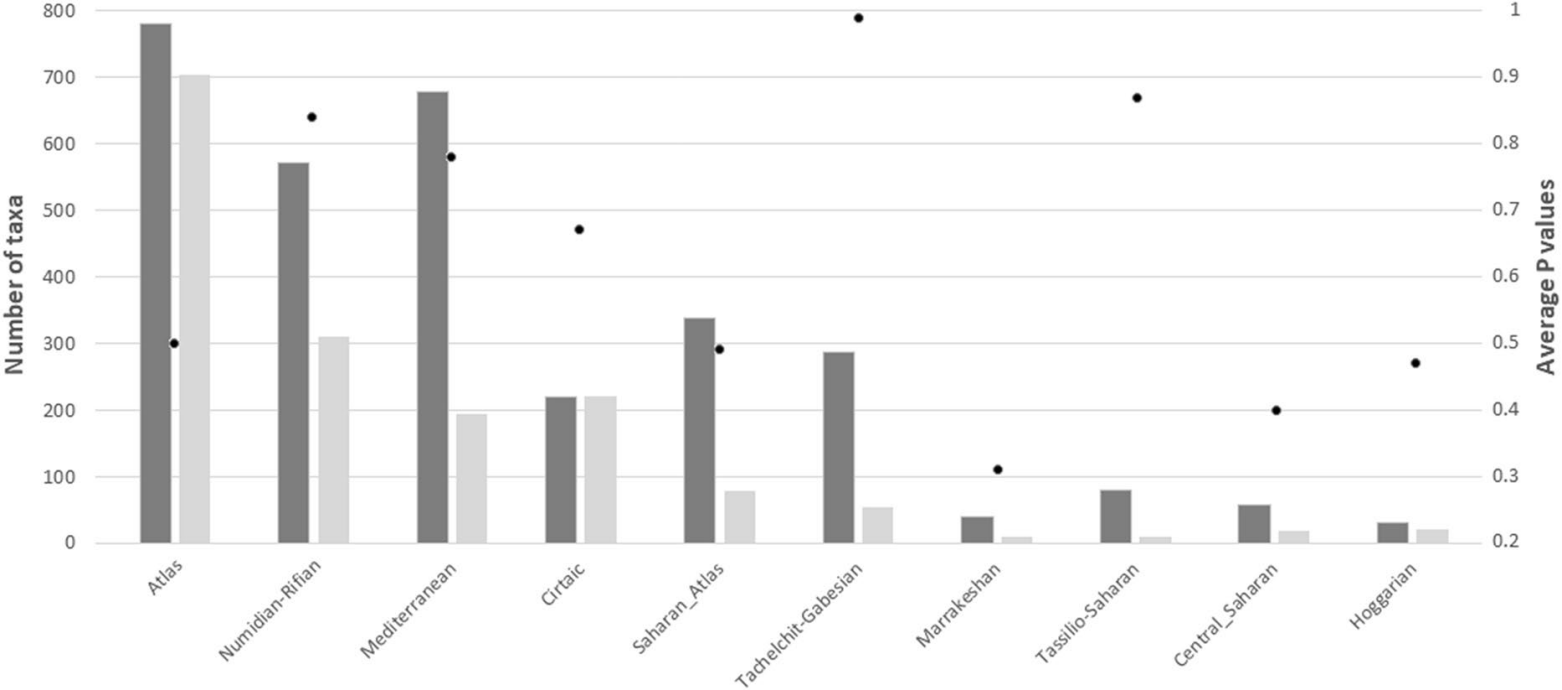
Number of all taxa (dark bars), number of taxa with highest indicator value in a particular cluster (light bars), and average *p*-value of all indicator taxa (black dots)

Results from IndVal analysis showed the exceptional richness of Atlas. For this cluster, 704 species had the highest indicator value, with a mean *p*-value of 0.50 (Fig. 4). Throughout the Maghreb, only 15 indicator taxa had a significant *p*-value (< 0.05). These taxa were present in the Numidian-Rifian and Mediterranean clusters (ten in Numidian-Rifian and five in Mediterranean). Analyses for a smaller number of clusters (from *K* = 2 to *K* = 10, according to hierarchical clustering) showed less than 10 significant indicator species. The correlation between the number of endemic taxa and cluster area is very weak (*R*^2^ = 0.0238), which is connected with a large area of desert clusters with a low number of taxa (Fig. S3).

## Discussion

The presented study considers various environmental factors to distinguish environmental clusters of the Maghreb, characterized by different conditions. Although the most important factors were climatic variables (temperature and precipitation), our approach differed from typical climatic classification, because of using additional information, such as habitat heterogeneity, insolation, and soil conditions. Thus, the results of our analysis provide more comprehensive information about the regionalization of the Maghreb area, and together with other data, such as the occurrence of endangered species or floristic ecoregions, can help to create effective environmental protection strategies. However, our model was rather simple and did not include many factors that could potentially change borders and the optimal number of clusters. It also did not include nearby areas like Libya, Western Sahara, and Mauritania, as well as the Tibesti Mountains in Northern Chad, which share many environmental and biological similarities with the analyzed countries. Additionally, the approach used in this study could not detect some small areas with clearly different environmental conditions, like oases or salt pans.

### Main drivers of regionalization

The variables used for regionalization differed significantly between estimated clusters. The most important were temperature amplitude (which is connected strongly with continentalism), precipitation seasonality, and precipitation in the warmest quarter. These three variables distinguished desert areas and regions with humid climates, which were shaped by proximity to the Mediterranean Sea and the Atlantic Ocean. Although the bioclimatic variables were the most important factors in the model, soil conditions and sun irradiation also had high significance, especially in the case of southern clusters. Habitat heterogeneity had the lowest impact on the model. The high influence of climatic conditions on environmental clustering and the low significance of vegetation parameters was also described for the bioregionalization of Egypt (Abdelaal et al., 2020).

### Endemic flora of the Maghreb

According to Médail and Quézel (1997), the entire Maghreb (Algeria, Morocco, and Tunisia) has 9,522 endemic plant taxa: 4,200 in Morocco (20% of all plant taxa in this country), 3,160 in Algeria (8%), and 2,162 in Tunisia (2%). Recent work “Index synonymique de la flore d’Afrique du Nord” (Dobignard & Chatelain, 2010–2013) mentioned different numbers of endemic plants occurring in each country: 5,354 are reported in Morocco, 4,449 in Algeria, and 2,828 in Tunisia with an endemism rate of 18.20%, 6.51%, and 2.60%, respectively. Among these taxa, several are common to all three countries. Especially rich are the Atlas Mountains and the Mediterranean coast of Maghreb. The higher number of endemic plants in mountainous and coastal regions was a common pattern, observed in other parts of the Mediterranean Basin (Abdelaal et al., 2020; Fenu et al., 2014; Lobo et al., 2001). The Maghreb had floristic similarities to the neighboring regions, both in Africa and Europe. Many plant species co-occurred in the southern part of the Iberian Peninsula and the Maghreb, especially in the Rif Mountains. The Gibraltar Strait was both a barrier and a core of biodiversity hotspot (Rodríguez-Sánchez et al., 2008).

The high level of endemism in the Mediterranean cluster was connected with many historical and environmental factors. During the glacial maximum, several refugia with mild climates in the Mediterranean Basin were a haven for many plant species; such refugia also occur in mountainous areas of North Africa (Médail & Diadema, 2009). Complex topography, typical for this region, modified the connectivity between these small areas. Isolation and restriction of gene flow favored adaptation and speciation (Thompson, 2005). Habitat conditions, like climatic and edaphic factors, can also shape adaptive divergence (Buira et al., 2021), especially in the case of relict and endemic species, which occur in small, isolated areas (Hermant et al., 2013; Kruckeberg & Rabinowitz, 1985). Suitable environmental conditions in the Mediterranean Basin caused this area to become one of the biodiversity “hotspot,” and the northern Maghreb coasts also harbor a large number of taxa. Much fewer plant species occur in southern areas of the Maghreb, as it is covered mostly by deserts. The Sahara experiences many wet and dry periods, which were also associated with changes in flora (Gasse, 2000; Tierney et al., 2017). The final formation of the desert climate in its present shape took place about 2700 cal yr B.P (Kröpelin et al., 2008). During the desertification, many plants were shifted into wetter areas in the northern Maghreb as well as in mountainous areas within the Sahara, such as Ahaggar and Tibesti (Boucheneb & Benhouhou, 2012). Future climate change, connected with higher temperature and lower precipitation, probably will be a big threat for many species of North Africa. The most threatened species are those that occur in a small, limited area where changes in environmental conditions can alter suitability. For this reason, ex situ conservation programs are emerging for endemic species (Libiad et al., 2020).

### Characterization of environmental clusters

The hierarchical clustering according to endemic plant occurrences allows merging estimated clusters into three main groups, similar to the results of environmental clustering for *K* = 3. The first group consisted of the Atlas cluster, the second was made up of two northern clusters (Numidian-Rifian and Mediterranean), and the third consisted of the remaining eight clusters, characterized by a more dry climate.

The Atlas cluster (1) with an average altitude of 1721 m a.s.l. comprises mountainous areas from the Anti-Atlas in the south, throughout the chains of the High and Middle Atlas Mountains to the western part of Tell Atlas in north Algeria. It is the coldest area in the Maghreb, with an annual mean temperature of 13.53 °C and the lowest temperature of the coldest month below 0 °C (− 1.02 °C). This cluster has rather high precipitation, similar to the northern, coastal areas. Mediterranean climate types prevail, with cold-semi arid climates in the southern and eastern parts. Mediterranean forests cover a large part of this cluster; one ecoregion – Mediterranean High Atlas juniper steppe, which appears in the highest elevations – is endemic to the Atlas. Mountainous areas are characterized by very high biodiversity (Bouchet et al., 2018). Many plant species occur only here, for example, *Cupressus atlantica* Gaussen (Sękiewicz et al., 2020) and critically endangered *Romulea villaretii* Dobignard. Totally, 297 plant taxa are endemic to Atlas cluster; two genera: *Aliella* (5 species) and *Heliocauta* (1 species) occur only in this mountainous region. Thus, this cluster formed its own group in hierarchical analysis according to endemic taxa, had the highest number of potential indicator taxa and highest value of the *α*-index.

Along the northern coasts of Maghreb, two clusters were distinguished: Numidian-Rifian (2), which consisted of the Rif Mountains in Morocco together with coastal areas in eastern Algeria and western Tunisia, and the Mediterranean (3), which was divided into two subclusters - Atlanto-Mediterranean in north Morocco and northwest Algeria and Carthaginian in north Tunisia. The northern part of Maghreb hosts rich biodiversity; many glacial refugia are located in mountainous and coastal areas (Médail & Diadema, 2009). Three genera are endemic for this region: *Fezia*, *Kremeriella*, and *Rytidocarpus.* Both clusters are covered by Mediterranean woodlands and forests and Mediterranean conifer and mixed forests. The climate is mostly the hot-summer Mediterranean, with some areas of semi-arid and desert climates in the southernmost parts of the Mediterranean cluster in Morocco. The Numidian-Rifian cluster with mean annual precipitation above 700 mm is the wettest environmental cluster. It is also characterized by the lowest potential evapotranspiration, sun irradiation, isothermality, and the mean diurnal temperature range. Soils have a low pH and high carbon content; edaphic conditions clearly distinguish this cluster from the others. Such suitability of soil and climate had a positive influence on plant growth, resulting in the highest values of both Evenness and Diversity of Enhanced Vegetation Index, as well as in a high number of endemic species. Several rare species occur only in the Numidian-Rifian cluster (like critically endangered *Abies numidica* de Lannoy ex Carrière, *Rumex tunetanus* Barratte et Murb., and *Vicia fulgens* Batt.). In terms of the number of endemic plant taxa per area, the Rifian subcluster is extremely rich, with 58 taxa occurring only in this subcluster, for example, *Abies marocana* Trab. Together with the Betic region in southern Spain, the Rif mountains form a biodiversity hotspot. The human impact in this area was surprisingly low, which allowed for the preservation of a large part of biodiversity (Cheddadi et al., 2016; Muller et al., 2015). The Mediterranean cluster has similar flora to that of the Numidian-Rifian; however, the precipitation is much lower, and the soils are poorer. The Atlanto-Mediterranean subcluster has the lowest temperature amplitude (mean diurnal range of temperature 6.93°) as well as the lowest isothermality (29.93%). Two species endemic to this subcluster are critically endangered: *Juncus maroccanus* Kirschner and *Pulicaria filaginoides* Pomel.

In the southern part of Maghreb, the climate is much drier, while the soil is rather poor; thus, plant diversity is lower. This area was divided into eight clusters (4–11), from which four are dominated by dry woodlands and steppe (clusters 4–7) and four are covered by deserts and xeric vegetation (clusters 8–11). The most northern of these clusters is the Cirtaic (4), which showed some similarities in terms of environmental conditions with Atlas (Fig. 3B). This area is dominated by mountains (Aures Mountains in the east, the Saharan Atlas in the west, and Tell Atlas in the north), albeit much lower than in the Atlas cluster (mean altitude in the Cirtaic cluster is 889 m a.s.l.). The climate in this area is cold and semi-arid, characterized by the lowest precipitation seasonality, with high precipitation in the driest quarter and average precipitation in the wettest quarter. Precipitation decreases in the southern part, where steppes replace Mediterranean vegetation (Habib et al., 2020). South to the Cirtaic, the Saharan Atlas cluster (5) is located. It is a large zone (315,200.08 km^2^) covered mostly by dry woodlands and steppes, which in the southern part turns into xeric steppes and woodlands (Taibaoui et al., 2020). The climate here is desert, and soils, mostly calcisols and leptosols, have a low carbon content, which distinguishes this cluster from the Cirtaic. However, Saharan Atlas shows some floristic similarities to the northern clusters, for example, large numbers of species from genera *Centaurea* and *Teucrium*. Another cluster with intermediate environmental conditions is Tachelchit-Gabesian (6), which consists of three subclusters that are geographically distant: first (6a) on the south coast of Morocco, second (6b) in the valley of the Moulouy River near city Taourirt, and third (6c) in the south coast of Tunisia. Although these areas are located close to the sea, the climate there is much drier than in the Numidian-Rifian and Mediterranean clusters; however, the temperature amplitude is similar, and the potential evapotranspiration seasonality much lower. Further differences were soil conditions: carbon content in the Tachelchit-Gabesian cluster is low, which is connected with poorer vegetation. However, many rare plants occur in this cluster, for example, *Lotus pseudocreticus* Maire & al. The core range of an economically important endemic species, *Argania spinosa* (L.) Skeels, is located in the 6a subcluster (Alba-Sánchez et al., 2015). Results of environmental clustering suggested that *Argania* could also grow in southern Tunisia, in subcluster 6c. However, previous analyses of the theoretical range of this species were focused on the natural range in Morocco, and its suitability in other countries is unknown (Alba-Sánchez et al., 2015; Moukrim et al., 2018, 2019). Two genera, *Hannonia* and *Ismelia*, are endemic to subcluster 6a. Subcluster 6b is of particular interest because it is a small area containing the valley with dry, hot desert conditions surrounded by a much more suitable Mediterranean cluster. Located in a small, coastal area of south Tunisia subregion 6c, although having similar environmental conditions as 6a and 6b, is characterized by a low number of endemic plant species (Fig. S3). The Marrakeshan cluster (7) has also a small area; it is situated at the foot of the Atlas Mountains around Marrakech city. Environmental conditions in this cluster are intermediate, with similarities both to northern clusters (3 and 4), with which it forms an environmental group (Fig. 3B), as well as subcluster 6a, which is also characterized by a high temperature and poor soils. Such mixed conditions were probably the reason for the separation of this small area as a separate cluster.

The southernmost part of Maghreb is characterized by a hot desert climate and very poor soils. The Tassilio-Saharan cluster (8), which borders Central Sahara on the west (8a subcluster) and south-east (8b subcluster), has the lowest annual temperature and highest diurnal temperature range. This area is intermediate between the hyper-arid desert and more humid mountainous areas of Ahaggar and Anti-Atlas. Similar environmental borders of climatic regions around central Sahara were obtained during the clustering of arid areas of Sahara-Sahel by principal component analysis (Brito et al., 2014). Subcluster 8a shows floristic similarity to the Tachelchit-Gabesian cluster, whereas in subcluster 8b there are many endemic plants that occur in the Hoggarian (10) cluster. Also, natural populations of *Cupressus dupreziana* A.Camus, one of the rarest gymnosperm species in the world, occur only in the Tassili mountains located in this subcluster (Sękiewicz et al., 2018). The largest part of southern Maghreb comprises the Central Saharan cluster (9), which covers an area of 1,334,674.27 km^2^ and has the highest value of temperature seasonality, highest maximum temperatures, and lowest precipitation in the warmest quarter. This hot, hyper-arid center of the desert is formed by rocky plains, ergs, dry valleys, and plateaus; vegetation is very limited. Potential evapotranspiration in the Central Sahara cluster is very high, which, combined with low precipitation, is a major constraint on plant growth. Despite these harsh conditions, some plants occur only in this cluster (for example *Calligonum calvescens* Maire, *Helianthemum eriocephalum* Pomel, *Salvia pseudojaminiana* Chevall, and *Salicornia deserticola* A. Chev.). South of the central Sahara are the Ahaggar Mountains, which are well-known because of the abundance of many relict populations of Mediterranean flora, such as *Olea europaea subsp. laperrinei* (Batt. & Trab.) Cif. and *Myrtus nivellei subsp. nivellei* Batt. & Trab. (Besnard et al., 2007; Boucheneb & Benhouhou, 2012; Migliore et al., 2013). These mountains, with the highest peak, Mount Tahat at 2,908 m, form the Hoggarian cluster (10). This area is colder and more humid than other southern regions of the Maghreb. Additionally, cold quarters are much drier than those that are warm, which distinguishes this cluster from all others except Tannezouft (11). The number of Maghrebian endemic taxa is quite low in the Ahaggar, but there are many species (not included in our list) that co-occur in this region and the Tibesti Mountains in northern Chad. The southernmost cluster of Tannezouft (11) is hot and dry, dominated by the South Saharan ecoregion, which in the deep south turns into an acacia savanna. A specific feature of this area is very high precipitation seasonality. No endemic plants typical for the Maghreb occur in this area; this may be due to poor description of this remote zone in literature, as well as the border location of this area, which may refer biologically to the Sahel.

## Conclusions

Our work is a contribution to the biogeography of North Africa. Eleven environmental clusters were estimated according to various environmental variables, providing a simple basis for understanding the spatial patterns of the environment in this area. Knowing the boundaries of areas with different conditions can be especially important in the face of climate change that can lead to the expansion of desert areas from the south to the north. A list of endemic plant species designed for this analysis together with environmental data will facilitate the planning of future research in the Maghreb, arranging methods of biodiversity protection, and the development of better, more detailed regionalization. Furthermore, this data would be helpful in determining the most important regions for future conservation programs.

## Supplementary information

Below is the link to the electronic supplementary material.

Supplementary file1 (TIFF 150 KB)

Supplementary file2 (TIFF 12107 KB)

Supplementary file3 (TIFF 181 KB)

Supplementary file4 (XLSX 38 KB)

Supplementary file5 (XLSX 175 KB)

Supplementary file6 (XLSX 10 KB)

Supplementary file7 (DOCX 18 KB)

## Data Availability

List of endemic species and relevant information about environmental variables are included as a supplementary material.
