# Supplementary figures and images for: Environmental regionalization and endemic plant distribution in the Maghreb
Source: Environ Monit Assess. 2022 Jan 15;194(2):100. doi: 10.1007/s10661-021-09707-6 (PMC8761123; doi:10.1007/s10661-021-09707-6)

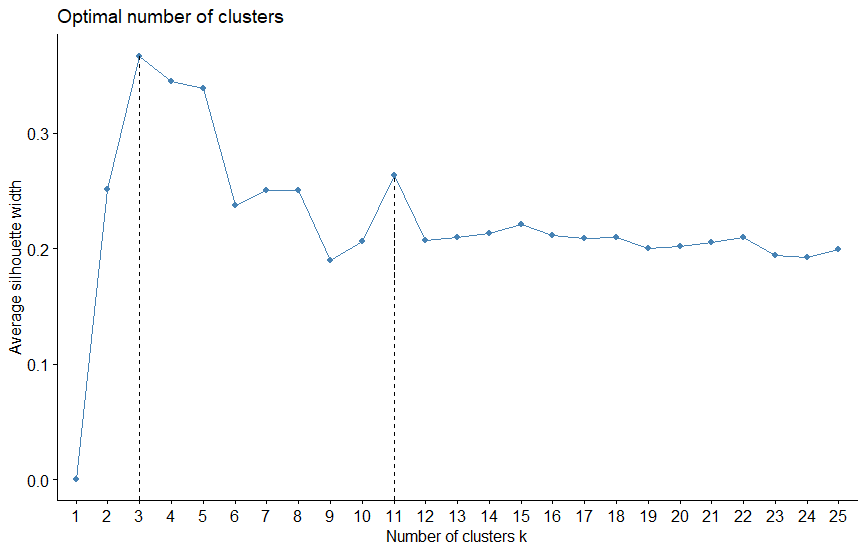

Supplement: Supplementary file 1 — Supplementary file1 (TIFF 150 KB) [file 10661_2021_9707_MOESM1_ESM.tiff]

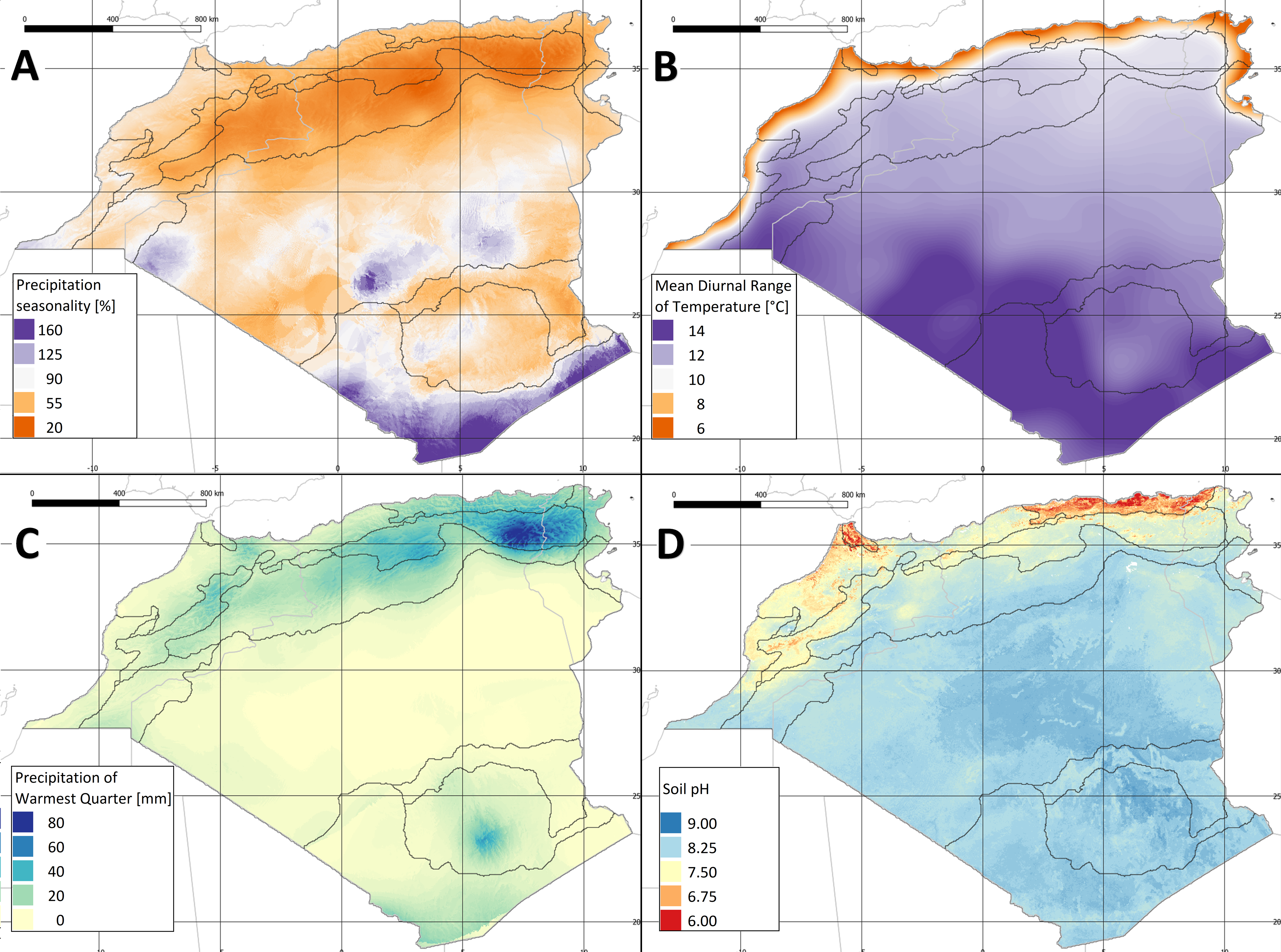

Supplement: Supplementary file 2 — Supplementary file2 (TIFF 12107 KB) [file 10661_2021_9707_MOESM2_ESM.tiff]

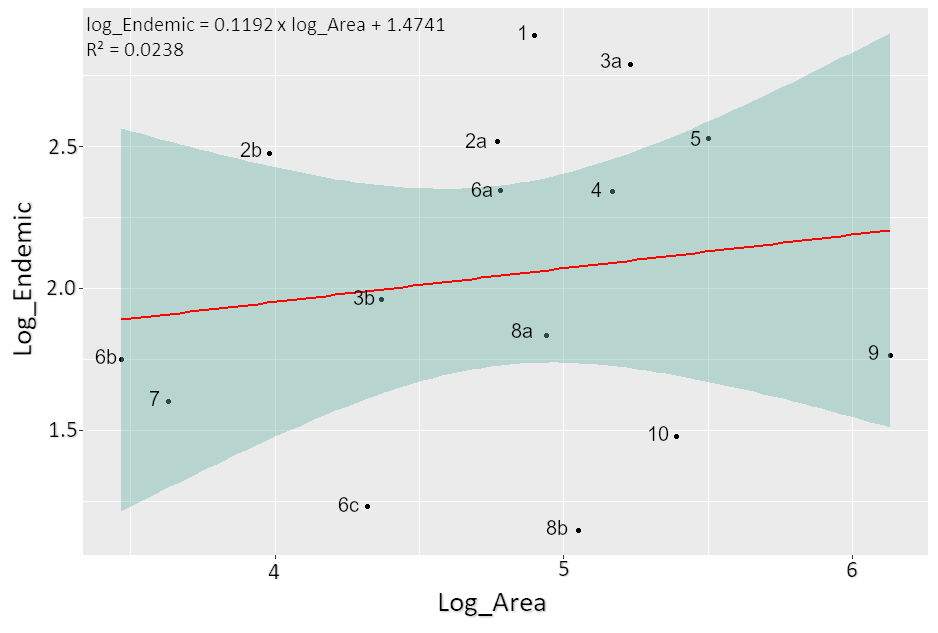

Supplement: Supplementary file 3 — Supplementary file3 (TIFF 181 KB) [file 10661_2021_9707_MOESM3_ESM.tiff]
