# Supplementary material for: Environmental regionalization and endemic plant distribution in the Maghreb
Source: Environ Monit Assess. 2022 Jan 15;194(2):100. doi: 10.1007/s10661-021-09707-6 (PMC8761123; doi:10.1007/s10661-021-09707-6)
Supplement: Supplementary file 7 — Supplementary file7 (DOCX 18 KB) [file 10661_2021_9707_MOESM7_ESM.docx]

Table S3. Endemic genera of Northwest Africa.

| Genera | Family | N | Regions of occurrence |
| --- | --- | --- | --- |
| *Agropyropsis* (Batt. & Trab.) A.Camus | Poaceae | 1 | 3a, 4 |
| *Aliella* M. Qaiser et H. W. Lack | Asteraceae | 5 | 1 |
| *Argania* Roem. & Schult | Sapotaceae | 1 | 3a, 6a, 8a |
| *Argyrocytisus* (Maire) Frodin & Heywood ex Raynaud | Fabaceae | 1 | 1, 2b, 3a |
| *Ceratocnemum* Coss. & Balansa | Brassicaceae | 1 | 1, 3a |
| *Cordylocarpus* Desf. | Brassicaceae | 1 | 2a, 3a, 6b |
| *Crambella* Maire | Brassicaceae | 1 | 3a, 6b |
| *Feeria* (Schousb.) Buser | Campanulaceae | 1 | 1, 3a |
| *Fezia* Pit. ex Batt. | Brassicaceae | 1 | 3a |
| *Foleyola* Maire | Brassicaceae | 1 | 5, 8a, 9 |
| *Hannonia* Braun-Blanq. & Maire | Amaryllidaceae | 1 | 6a |
| *Heliocauta* Humphries | Asteraceae | 1 | 1 |
| *Hemicrambe* Webb | Brassicaceae | 1 | 2b |
| *Hesperolaburnum* Maire | Fabaceae | 1 | 1, 6a |
| *Ismelia* Cass. | Asteraceae | 1 | 6a |
| *Kremeriella* Maire | Brassicaceae | 1 | 3a |
| *Mecomischus* Coss. ex Benth. & Hook.f. | Asteraceae | 2 | 3a, 4, 5, 6b |
| *Nivellea* B.H.Wilcox, K.Bremer & Humphries | Asteraceae | 1 | 1, 3a |
| *Oreobliton* Durieu & Moq. | Amaranthaceae | 1 | 2a, 4 |
| *Otocarpus* Durieu | Brassicaceae | 1 | 2a, 3a, 4 |
| *Pseudoridolfia* Reduron, Mathez & S.R.Downie | Apiaceae | 1 | 1, 3a |
| *Psychine* Desf. | Brassicaceae | 1 | 1, 2b, 3a, 4, 5, 6a, 7 |
| *Raffenaldia* Godr. | Brassicaceae | 2 | 1, 2b, 3a, 4, 5 |
| *Rhetinolepis*  Coss. | Asteraceae | 1 | 5, 8a, 9 |
| *Rhodanthemum* B.H.Wilcox, K.Bremer & Humphries | Asteraceae | 13 | 1, 2b, 3a, 5, 6a, 7, 8a |
| *Rytidocarpus* Coss. | Brassicaceae | 1 | 3a |
| *Saccocalyx* Coss. & Durieu | Lamiaceae | 1 | 4, 5 |
| *Selinopsis* Coss. & Durieu ex Batt. | Apiaceae | 2 | 1, 2a, 4 |
| *Trachystoma* O.E.Schulz | Brassicaceae | 3 | 1, 3a, 7 |
| *Traganopsis* Maire & Wilczek | Amaranthaceae | 1 | 6a, 8a |
| *Warionia* Benth. & Coss. | Asteraceae | 1 | 1, 3a, 5, 6a, 7, 8a, 9 |

N - number of species. Regions of occurrence:, 1 – Atlas, 2a – Numidian, 2b - Rifian, 3a – Atlanto-Mediterranean, 4- Cirtaic, 5 – Saharan Atlas, 6a – Tachelhit, 6b – Taurirt, 7 – Marrakeshan, 8a – Western Saharan, 9 – Central Saharan
